# Predictive Immunohistochemistry in Cholangiocarcinoma: Current Clinical Utility, Practical Limitations, and Emerging Directions

**DOI:** 10.1111/apm.70230

**Published:** 2026-06-08

**Authors:** Francesco Vasuri, Alessandra Boccaccino, Stefano Tamberi, Luca Saragoni

**Affiliations:** ^1^ Pathology Unit Santa Maria Delle Croci Hospital Ravenna Italy; ^2^ Department of Medical and Surgical Sciences (DIMEC) University of Bologna Bologna Italy; ^3^ Oncology Unit Santa Maria Delle Croci Hospital Ravenna Italy

**Keywords:** cholangiocarcinoma, claudin 18.2, HER2, immunohistochemistry, mismatch repair, PD‐L1

## Abstract

Cholangiocarcinoma (CCA) remains a difficult‐to‐treat biliary malignancy in which therapeutic stratification increasingly depends on predictive biomarkers. Although next‐generation sequencing is essential for the detection of targetable genomic alterations, immunohistochemistry (IHC) retains a practical role because it is widely available, rapid, and tissue‐sparing. This narrative review summarizes the current and emerging role of predictive IHC in CCA, with emphasis on its clinical utility, interpretative pitfalls, and integration with molecular testing. HER2 and mismatch repair proteins currently represent the most relevant IHC‐based markers in routine practice, albeit in selected subsets of patients. By contrast, PD‐L1 has clear biological relevance but limited value as a stand‐alone treatment selector in CCA, whereas Claudin 18.2 is promising but still investigational. Additional lines of research, including tumor microenvironment profiling, integrin‐related pathways, and other theragnostic targets, may further refine patient selection, but these approaches are not yet standardized. Digital image analysis, radiomics, and machine learning are likely to improve quantification and may support future biomarker integration. A practical pathology‐oriented approach should prioritize tissue stewardship, conservative interpretation of IHC results, and close coordination with molecular methods.

## 1 Introduction

Cholangiocarcinoma (CCA) is a heterogeneous group of aggressive malignancies arising from the intrahepatic and extrahepatic biliary epithelium. In Western countries, incidence and mortality remain clinically relevant, and the prognosis is still poor because many patients present with advanced disease, develop early relapse, or derive limited benefit from later treatment lines [1]. During the last few years, the addition of immune checkpoint inhibition to first‐line chemotherapy has improved outcomes in advanced biliary tract cancer, but this has not eliminated the need for biomarker‐driven treatment strategies after progression or in selected molecular subsets [2, 3].

Current precision oncology in CCA is still driven mainly by molecular alterations such as FGFR2 fusions and IDH1 mutations, which are enriched in selected subtypes, especially small‐duct intrahepatic CCA (iCCA). Nevertheless, compared with other solid tumors, the number of immediately actionable alterations remains limited, and many patients still lack an effective biomarker‐guided therapeutic option after first‐line treatment [4]. In this setting, the pathologist retains a central role in tissue triage and in the assessment of protein biomarkers that can be evaluated rapidly by IHC on routine formalin‐fixed paraffin‐embedded material.

IHC is particularly attractive in CCA because samples are often small, fragmented, or obtained from anatomically challenging sites, and turnaround time matters when treatment decisions depend on scarce tissue. In other tumor types, automated IHC can complement or occasionally substitute for molecular methods in selected contexts, provided that assay performance and interpretation are standardized [5]. In CCA, however, the evidence is more uneven, and not all markers with biological appeal have the same degree of analytical maturity or therapeutic actionability.

## 2 Scope and Literature Focus

**FIGURE 1:**
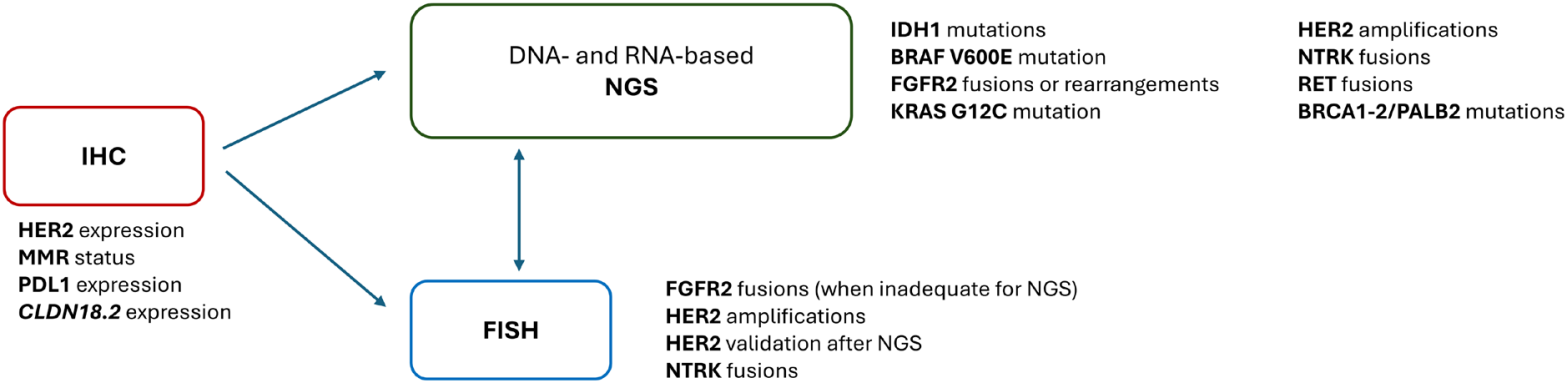
Practical laboratory workflow including the main applied or potential immunohistochemical (IHC), fluorescence in situ hybridization (FISH), and next‐generation sequencing (NGS) predictive markers in cholangiocarcinoma. The present review focuses on IHC markers alone.

This review is aimed at distinguishing markers that already have practical clinical relevance from those that are biologically interesting but still investigational. The focus is on predictive IHC markers that are already used, likely to be used in the near future, or relevant as screening tools that should be interpreted in conjunction with confirmatory molecular testing. IHC markers are considered as the beginning of a predictive workflow, starting from the pathology laboratory, but including also other techniques, as illustrated in Figure 1.

This manuscript is conceived as a narrative review of the English‐language literature, centered on human studies and clinically oriented translational work relevant to tissue predictive biomarkers in CCA. During preparation of this review, the authors used Consensus to assist with literature discovery and preliminary thematic exploration of peer‐reviewed publications. Queries from PubMed were performed using the keywords “predictive markers”, “tissue markers”, “immunohistochemistry”, and “cholangiocarcinoma”, followed by specific queries for every single emerging topic. The authors independently reviewed the original articles, verified all factual statements, selected the references, and took full responsibility for the final content of the manuscript. Priority is given to studies that address prevalence, assay interpretation, potential treatment implications, and pathology workflow (Table 1). Experimental studies are discussed when they help clarify biological rationale or indicate near‐term translational relevance. Papers addressing biological markers in CCA without pathological tissue (and especially immunohistochemical) applications (e.g., liquid biopsy, pure molecular markers) were excluded.

**TABLE 1:** Practical summary of the main IHC‐based predictive markers in cholangiocarcinoma.

Marker	Prevalence in CCA (site‐specific)	Staining pattern	Current practical role	Main pathology issue/confirmation	Current status
HER2	Variable: 3% in iCCA and perihilar, 18% in eCCA and up to 31% in GBC [6]	Complete or basolateral/lateral membranous; external control needed	Screening marker for selected advanced cases with potential anti‐HER2 therapy (available Zanidatamab, Trastuzumab‐Deruxtecan) [7]	No fully standardized CCA‐specific scoring; small biopsies and equivocal staining may require confirmation by in situ hybridization [7, 8]	Clinically relevant now—Phase II Useful for a screening/stratification assay, but not yet as stand‐alone marker in CCA
MMR proteins	Rare (about 1%–6%), regardless of the tumor site [9, 10]	Loss of nuclear expression in tumor cells defines dMMR; internal controls usually present	Screening for dMMR/MSI‐H cases with immunotherapy implications (Pembrolizumab) [4, 11]	Low prevalence; internal controls and adequate fixation are essential [9, 10]; dMMR may require MSI molecular confirmation	Clinically relevant now—Phase II Rare, but with substantial therapeutic implications
PD‐L1	Less related to site or histotype; more dependent on the immune microenvironment (tumor‐infiltrating lymphocytes, macrophages) [12, 13, 14, 15, 16]	Membranous staining in tumor and/or immune cells; no validated positivity assessment system in CCA	Biologically informative, but not a reliable stand‐alone treatment selector (available Pembrolizumab or Durvalumab *plus* cisplatin‐gemcitabine) [17, 18, 19, 20, 21, 22, 23, 24, 25, 26]	Assay heterogeneity and lack of validated CCA‐specific scoring [18, 24, 25, 26]	Limited routine utility—Phase III
CLDN18.2	Minority of cases overall, more in eCCA, GBC and large‐duct iCCA, than small‐duct iCCA [27, 28, 29]	Membranous staining; interest focused on strong expression levels and percentage of positive tumor cells	Research and trial‐enrichment marker	Cut‐offs and therapeutic validation in CCA are still incomplete [30]	Emerging/investigational; not ready for routine pathology workflow

Abbreviations: eCCA, extrahepatic cholangiocarcinoma; GBC, gallbladder carcinoma; iCCA, intrahepatic cholangiocarcinoma.

## 3 Current Clinically Relevant IHC Markers in CCA

### 3.1 HER2

Among the currently available protein targets assessable by IHC, HER2 is the marker with the clearest potential for clinically actionable stratification in selected CCA subsets. HER2 is a well‐established prognostic and predictive biomarker in breast and gastric cancer, and analogous interest has progressively extended to biliary tract malignancies [6]. In CCA, HER2 overexpression and/or amplification has been associated with adverse clinicopathologic features and has been reported in a minority of cases, with frequencies ranging broadly from approximately 3%–18% depending on the cohort, anatomical site, and scoring strategy [6, 7]. Hiraoka et al. found a 3%–4% prevalence of positive cases among iCCA and perihilar CCA, 18.5% among distal extrahepatic CCA (eCCA), and more than 30% among gallbladder carcinomas (GBC) [6].

From a practical standpoint, this site‐related distribution matters because pre‐treatment sampling in extrahepatic lesions is often suboptimal. Small biopsies, low neoplastic cellularity, crush artifact, and limited tissue reserve can all compromise reliable preoperative assessment [8]. In addition, CCA‐specific scoring criteria are not standardized as in breast or gastric pathology, and direct transfer of scoring systems across tumor types may lead to over‐ or under‐calling of equivocal cases. Accordingly, HER2 IHC should be used as a screening and stratification tool, not as an isolated decision point detached from morphology, sampling quality, and confirmatory testing.

Despite these limitations, the therapeutic rationale for HER2 assessment is stronger than for most other IHC markers in CCA. Preclinical evidence on cell lines showed that Trastuzumab can inhibit CCA growth and modulate cytotoxicity through multiple mechanisms, including antibody‐ and complement‐mediated effects, even in models with relatively low immunoreactivity [31]. Interestingly, in the same paper the authors found a higher HER2 expression in liver fluke‐associated CCA cells compared to others, suggesting a potential etiology‐dependent difference [31]. On clinical grounds, multiple phase II studies have explored anti‐HER2 strategies in pretreated biliary tract cancer. Reported response rates are variable but often meaningful, even if durability and survival endpoints are not uniformly robust across studies [7]. Notably, Zanidatamab in HERIZON‐BTC01 and Trastuzumab‐Deruxtecan in DESTINY‐PanTumor02 have strengthened the relevance of HER2‐positive biliary tumors (defined as both HER2 2+ and 3+ positivity in both trials) as a therapeutically distinct subgroup [32, 33].

For daily practice, the most defensible interpretation is that HER2 IHC already has a practical role in advanced CCA, especially in extrahepatic and gallbladder‐associated cases, but only within an integrated algorithm. Strong membranous staining in an appropriate clinical setting should prompt either confirmatory in situ hybridization or correlation with molecular profiling, whereas weak or equivocal staining requires particular attention. In this sense, HER2 is best regarded as the most immediately useful IHC‐based predictive marker in CCA, but not yet as a fully standardized stand‐alone companion diagnostic.

### 3.2 Mismatch Repair Proteins

Mismatch repair (MMR) deficiency is uncommon in CCA, but its clinical significance is disproportionate to its prevalence because deficient mismatch repair (dMMR) and microsatellite instability‐high (MSI‐H) status identify tumors that may derive major benefit from immune checkpoint blockade [4, 11]. As in colorectal and endometrial pathology, IHC for MLH1, PMS2, MSH2, and MSH6 is a practical, accessible, and tissue‐efficient way to screen for this biology in routine practice. The principal limitation is the rarity of dMMR cases among CCA: in a Western study on a tissue micro‐array built with 308 CCA, only 4 (1.3%) were MSI‐H, 2 iCCA and 2 perihilar CCA, all with “atypical” histological features, such as papillary, mucinous and/or cribriform histology [9]. A subsequent study based on IHC reported dMMR in approximately 6% of cases and confirmed a higher prevalence of dMMR cases in unusual CCA histotypes such as mucinous or *signet ring* tumors [10]. These prevalence differences almost certainly reflect cohort composition, methodology, and the low absolute number of positive cases, but both studies support the same practical conclusion: the finding of dMMR is infrequent among CCA, yet clinically relevant enough that it should not be ignored.

Pembrolizumab has agnostic approval for dMMR/MSI‐H malignancies, including biliary tumors, which makes MMR IHC one of the few low‐cost assays with potentially direct therapeutic consequences in CCA [4, 11]. The fact that chemoimmunotherapy is now used broadly in the first‐line metastatic setting does not eliminate the value of identifying dMMR, because this information may still influence later‐line strategies, clinical‐trial eligibility, and the interpretation of exceptional responses. The assay is also technically familiar to most pathology laboratories. The interpretation, however, remains dependent on fundamentals such as adequate fixation, sufficient viable tumor, and internal non‐neoplastic controls. In small biopsies or poorly fixed samples, aberrant or patchy staining can be misleading, and equivocal findings should prompt orthogonal confirmation by MSI testing when feasible. Overall, while dMMR is rare in CCA, MMR IHC remains one of the most clinically meaningful predictive tests that a routine pathology laboratory can provide.

## 4 Markers With Limited or Emerging Predictive Utility

### 4.1 PD‐L1

PD‐L1 is one of the most intensively discussed biomarkers in CCA, but also one of the most difficult to position correctly in routine practice. Biologically, PD‐L1 expression reflects an active interaction between tumor cells and the immune microenvironment. In CCA, however, the cells most often expressing PD‐L1 are tumor‐associated macrophages and lymphocytes rather than the neoplastic epithelial component itself [12, 13, 14, 15, 16]. This distinction is not merely descriptive: it directly affects scoring, reproducibility, and the meaning of a “positive” result. In several studies, PD‐L1 expression in the immune compartment correlates with more aggressive disease and with specific immune phenotypes, particularly in intrahepatic CCA [12, 13, 14]. It must be kept in mind that, because PD‐L1 is mostly expressed by inflammatory cells, these papers focused on the PD‐L1‐positive inflammatory populations, rather than CCA subtype or location. For example, Walter et al. [34] found PD‐L1 positivity in 12% of eCCA tumor cells and in 30% of tumor‐associated macrophages. Evidence indicates that bona fide PD‐L1 positivity in tumor cells is uncommon among all primary liver as well as biliary cancers, and when present it is often associated with coexisting inflammatory infiltrates rather than a stable tumor‐cell dominant pattern [15, 16]. These observations fit the broader biology of liver tumors but complicate the use of PD‐L1 as a standard predictive assay because different studies count different cellular compartments and use different thresholds.

This analytical heterogeneity is a major problem. Published series have used tumor‐area proportion, combined positive score, percentage of positive tumor cells, and other non‐standardized methods. As a result, PD‐L1 IHC has not demonstrated a clear and reproducible ability to identify which CCA patients will benefit most from PD‐1/PD‐L1‐directed therapy [17, 18, 19, 20, 21, 22, 23, 24, 25, 26]. This is especially relevant now that first‐line chemoimmunotherapy combinations such as Durvalumab or Pembrolizumab with Gemcitabine‐cisplatin are used without PD‐L1‐based selection with benefits for patients' survival, as it is the case of the KEYNOTE‐966 and the TOPAZ‐1 trials, as well as other studies [2, 3, 20, 21, 22, 23].

For this reason, PD‐L1 should not be presented as a routine predictive marker with the same weight as HER2 or MMR status in CCA. Its role is better defined as biologically informative and potentially useful in translational stratification, especially when interpreted together with the broader immune contexture. In routine diagnostic reporting, however, PD‐L1 remains a context‐dependent and non‐standardized marker rather than a reliable stand‐alone selector.

### 4.2 Claudin 18.2

Claudin 18.2 (CLDN18.2) has emerged as an attractive membrane target in gastrointestinal oncology because it can be assessed by IHC and has already acquired predictive relevance in gastric and gastro‐esophageal adenocarcinoma treated with zolbetuximab [27]. This has naturally generated interest in CCA, a disease in which additional druggable markers are needed.

Available studies suggest that strong CLDN18.2 immunoreactivity is present in a minority of CCA: a recent tissue microarray‐based study by Kinzler et al. [28] found 13% of CCA to be positive, ranging from 7.4% of iCCA to 26.5% of perihilar CCA. The same authors found a positive correlation between CLDN18.2 positivity and tumor aggressiveness, such as perineural invasion and metastases [28]. More nuanced data indicate that prevalence is highly dependent on anatomical location and subtype, with lower expression in small‐duct intrahepatic CCA and substantially higher rates in extrahepatic and gallbladder‐associated carcinomas [29, 30]. This variability is important because it implies that CLDN18.2 should not be discussed as a uniform marker across all biliary tract sites. A recent Italian paper based on whole‐slide IHC rather than microarrays found even higher positive rates, from 8% of small‐duct iCCA to 33% of large‐duct iCCA to more than 50% of eCCA and GBC cases [30]. At present, the main appeal of CLDN18.2 in CCA is its translational promise rather than proven clinical utility. Early‐phase studies of anti‐CLDN18.2 strategies, including antibody‐drug conjugates, have shown activity signals, although response rates remain modest and data specific to advanced CCA are still preliminary. Moreover, the question of whether to apply the therapeutic cut‐off of 75% of positive cells (as used in gastric cancer) also to CCA remains to be demonstrated [30]. Additional experimental approaches, including nanotherapy‐based strategies, reinforce the biological plausibility of the target but do not yet establish a routine role for this assay [35].

Accordingly, CLDN18.2 should currently be framed as an emerging marker. It may help identify biologically distinct subsets and future trial candidates, particularly among extrahepatic or gallbladder‐related tumors, but assay cut‐offs, treatment relevance, and disease‐specific validation are still insufficient for routine predictive testing.

## 5 Other Predictive and Theragnostic Markers

### 5.1 Tumor Microenvironment as a Predictive Framework

The tumor microenvironment (TME) has become central to contemporary oncologic pathology because it has the potential to influence tumor progression, prognosis, and response to therapy in many solid tumor models. In CCA, immune cell density, composition, and spatial distribution appear to carry both biologic and potential therapeutic relevance. Conventional and multiplex IHC approaches have been used to characterize T‐cell subsets, regulatory lymphocytes, and checkpoint‐related immune populations, with markers such as CD3, CD4, CD8, FOXP3, and PD‐1 being among the most frequently investigated [36, 37, 38, 39, 40, 41]. A higher population of CTLA‐4‐positive and FOXP3‐positive lymphocytes has been shown to worsen the overall survival rate in CCA patients, especially in synergy with PD‐L1 co‐expression [36].

Several studies suggest that a higher density of CD4‐positive and/or CD8‐positive lymphocytes is associated with better survival, whereas increased FOXP3‐positive regulatory T‐cell infiltration correlates with nodal metastasis and adverse outcome [37, 38]. Beyond sheer quantity, spatial arrangement also appears informative. Intratumoral versus peritumoral localization, the presence of immune‐excluded patterns, and the distinction between “hot”, “ignored”, and “cold” phenotypes all seem to reflect biologically relevant differences that may influence therapeutic susceptibility [39, 40, 41]. A huge work by Ding et al. by means of Multiplex IHC on 962 Chinese cases found a relationship between intra‐ and peri‐tumoral tertiary lymphoid structures in CCA and prognosis, highlighting nearly 70% of 5‐year survival in the *immune‐active pattern* cases versus 3.4% of the *immune‐excluded pattern* cases [39].

These findings are conceptually important because they suggest that response prediction in CCA may ultimately require integrated tissue phenotyping rather than dependence on a single marker. TME may also be targetable: for example, CXCL9‐related signaling has been associated with increased survival and has been proposed as a potential lever to calibrate antitumor immune responses in intrahepatic CCA [42]. However, despite the promise of these observations, TME‐related IHC is not yet standardized for routine predictive use and currently remains primarily a translational research tool.

### 5.2 The Integrin Network and Additional Theragnostic Targets

Integrins represent another complex but potentially relevant family of transmembrane proteins in CCA. As adhesion molecules involved in cell–cell and cell‐matrix interactions, integrins have the ability to influence invasion, metastasis, matrix remodeling, and treatment resistance. Their dysregulation has been linked to poor prognosis in several malignancies, including CCA. Within this broad network, several subunits have been associated with aggressive behavior. ITGbeta6 was proposed as both a prognostic and predictive target because of its association with invasion, nodal metastasis, metalloprotease activation, and Rho family GTPase signaling [43]. Subsequent work linked overexpression of ITGbeta6 and alphavbeta6 to resistance to cisplatin‐based chemotherapy through MAPK pathway activation [44]. ITGalpha3 has likewise been associated with metastasis, poor outcome, and increased proliferation in vitro, while ITGbeta1 and aldehyde dehydrogenase‐related pathways have been implicated in cell proliferation and invasion [45, 46]. More recently, ITGbeta5 overexpression has also been reported as an adverse feature in a Chinese CCA cohort [47].

The main translational interest of integrins lies in the fact that anti‐integrin strategies already exist or are in development in other disease settings. This does not mean that a direct therapeutic transfer to CCA is imminent, because biology is highly context‐dependent and signaling is redundant, and many other steps are likely to be made for clinical practice. Even so, integrins remain a plausible bridge between prognostic IHC, mechanistic biology, and future target‐directed treatment development.

Other preclinical theragnostic candidates further illustrate how broad the field remains. Globo‐H, a carbohydrate antigen expressed by several epithelial cancers including CCA, has been explored as a target in experimental models. In a thioacetamide‐induced rat model of CCA, anti‐Globo‐H therapy suppressed tumor development and was associated with increased NK‐cell activity in the microenvironment [48]. Likewise, L1 cell adhesion molecule (L1CAM), a transmembrane glycoprotein linked to migration, progression, and resistance to apoptosis in CCA, has been explored in a preclinical in vitro model, with promising antitumor effects and applications in immune‐PET radioimmunotherapy [49]. These observations are intriguing, but they remain investigational and should not yet influence routine pathology reporting outside a research context.

### 5.3 Digital Pathology, Image Analysis, and Machine Learning

Quantitative image analysis and machine learning are increasingly relevant to predictive pathology because they can reduce interobserver variability, support reproducible scoring, and integrate histologic information with clinical and molecular data. In CCA, where several IHC markers are limited by heterogeneity and subjective thresholds, digital pathology may become particularly useful for more consistent biomarker assessment.

A further development is the radiomic prediction of tissue biomarkers using non‐invasive imaging. Early studies have explored whether imaging‐derived models can predict the expression of biologically important markers such as EGFR and VEGF, infer angiogenic characteristics, or estimate immune‐related features in CCA [50]. More specifically, radiomic models have been reported to predict PD‐1 and PD‐L1 expression, as well as survival, in intrahepatic CCA using combinations of MRI‐derived features and clinical variables [51].

At present, these approaches should be interpreted cautiously because validation, generalizability, and workflow integration remain limited. Even so, the field is moving quickly. Multi‐omics models that combine imaging, pathology, and molecular variables may eventually improve the prediction of morphological heterogeneity, inflammatory states, and even selected driver alterations such as IDH mutation [52, 53]. These methods are not ready to replace tissue‐based predictive testing, but they are likely to influence how future biomarker panels are quantified, integrated, and clinically deployed.

## 6 Conclusions

The current landscape of predictive IHC in CCA is promising but uneven. Not all markers discussed in the literature have the same level of clinical readiness, and a major practical task for the pathologist is to separate immediately useful assays from markers that remain exploratory. In routine practice, HER2 and MMR proteins currently represent the most defensible IHC‐based predictive tools because they can identify clinically relevant subgroups, even if both require careful interpretation and, in many cases, correlation with orthogonal testing [4, 6, 7, 9, 10, 11].

By contrast, PD‐L1 has clear biologic relevance but limited value as a stand‐alone selector in CCA because of substantial analytical heterogeneity and the absence of a validated disease‐specific scoring framework [12, 13, 14, 15, 16, 17, 18, 19, 20, 21, 22, 23, 24, 25, 26]. CLDN18.2 is a compelling emerging marker, particularly in selected anatomic subgroups, but it remains investigational until assay harmonization and therapeutic validation are more mature [27, 28, 29, 30, 35]. TME‐related phenotyping, integrin‐associated pathways, and preclinical theragnostic markers such as Globo‐H and L1CAM further expand the horizon of the field, yet they still belong mainly to translational pathology rather than routine decision‐making, albeit representing a stimulating perspective for future studies [36, 37, 38, 39, 40, 41, 42, 43, 44, 45, 46, 47, 48, 49].

The most realistic path forward is therefore not indiscriminate expansion of IHC panels, but smarter integration. Tissue stewardship, conservative interpretation of limited biopsies, and close coordination with molecular testing remain essential. As digital pathology, quantitative image analysis, radiomics, and machine learning evolve, future biomarker assessment in CCA will likely become more integrated and more precise. For now, however, the strongest contribution of IHC lies in focused, clinically disciplined use rather than in overextension of markers beyond the evidence currently available.

In current routine clinical practice, predictive IHC in CCA should be used selectively and within an integrated diagnostic workflow. HER2 and MMR assessment currently provide the most actionable information, particularly in advanced disease and in cases with appropriate clinicopathological features. PD‐L1 and CLDN18.2 should be further studied to gain useful application for oncologists, while tumor microenvironment and other pathobiological approaches remain mainly investigational. In the present as well as in the future, pathologists should always develop new IHC applications to be carried out in parallel with molecular testing to support clinically meaningful therapeutic options.

## Funding

The authors have nothing to report.

## Ethics Statement

The authors have nothing to report.

## Conflicts of Interest

The authors declare no conflicts of interest.

## Data Availability

Data sharing not applicable to this article as no datasets were generated or analysed during the current study.
